# ﻿Divergent altitudinal patterns of arbuscular and ectomycorrhizal fungal communities in a mid-subtropical mountain ecosystem

**DOI:** 10.3897/imafungus.16.e140187

**Published:** 2025-04-03

**Authors:** Taotao Wei, Huiguang Zhang, Shunfen Wang, Chunping Wu, Tieyao Tu, Yonglong Wang, Xin Qian

**Affiliations:** 1 College of Forestry, Fujian Agriculture and Forestry University, Fujian, China; 2 College of Life Sciences, Fujian Agriculture and Forestry University, Fujian, China; 3 Fujian Provincial Forestry Survey and Planning Institute, Fujian, China; 4 South China Botanical Garden, Chinese Academy of Sciences, Guangdong, China; 5 Baotou Teachers’ College, Baotou, 014030, China

**Keywords:** Altitudinal gradient, community structure, ecosystem multifunctionality, fungal diversity, Mycorrhizal fungi

## Abstract

Arbuscular mycorrhizal fungi (AMF) and ectomycorrhizal fungi (EMF) form ubiquitous symbiotic relationships with plants through co-evolutionary processes, providing multiple benefits for plant growth, productivity, health, and stress mitigation. Mountain ecosystem multifunctionality is significantly influenced by mycorrhizal responses to climate change, highlighting the importance of understanding the complex interactions between these fungi and environmental variables. In this study, we investigated five vegetation zones across an altitudinal gradient (675–2157 m a.s.l.) in Wuyi Mountain, one of the most well-preserved mid-subtropical mountain ecosystems in eastern China. Using high-throughput sequencing, we examined the altitudinal distribution patterns, community assembly mechanisms, and network interactions of soil AMF and EMF. Our analyses demonstrated significant altitudinal variations in the composition and diversity of mycorrhizal fungal communities. AMF richness peaked in the subalpine dwarf forest at intermediate elevations, whereas EMF richness was highest in the low-altitude evergreen broad-leaved forest, showing a marked decrease in the alpine meadow ecosystem. β-diversity decomposition revealed that species turnover constituted the primary mechanism of community differentiation for both fungal types, explaining >56% of the observed variation. Stochastic processes dominated community assembly, with the relative importance of dispersal limitation and drift showing distinct altitudinal patterns. Network analysis indicated that AMF networks reached maximum complexity in evergreen broad-leaved forests, while EMF networks showed similar complexity levels in coniferous forests. Among the examined factors, soil properties emerged as the predominant driver of altitudinal variations in ecosystem multifunctionality, followed by AMF communities and climatic variables. These findings provide critical insights into the ecological functions and environmental adaptations of mycorrhizal fungi, advancing our understanding of their responses to environmental changes in mountain ecosystems and informing evidence-based conservation strategies.

## ﻿Introduction

In terrestrial ecosystems, numerous fungal species establish intricate interactions with the root systems of various terrestrial plants, forming highly specialized and mutually beneficial symbiotic relationships known as mycorrhizae (Genre et al. 2020). These mycorrhizal fungi play a crucial role in maintaining ecosystem stability and functionality (van der Heijden et al. 2015; Weemstra et al. 2020; Bueno et al. 2021; Wu et al. 2022). Specifically, mycorrhizal fungi significantly contribute to multiple ecosystem processes, including soil primary productivity, nutrient cycling, water regulation and purification, carbon sequestration and climate regulation, pest and disease control, as well as pollutant degradation and detoxification (van der Heijden et al. 2015; Wang and Rengel 2024). Among the diverse mycorrhizal associations, AMF and EMF represent the most prevalent types. AMF form mutualistic associations with approximately 80% of terrestrial plant species, while EMF colonize nearly 60% of tree roots globally (Wang et al. 2006; Bonfim et al. 2016; Anthony et al. 2022). Notably, these two groups of mycorrhizal fungi exhibit distinct ecological functions and host specificities in their respective ecosystems.

In the mycorrhizal symbiotic system, vegetation maintains nutrient homeostasis by assimilating soil elements mobilized through mycorrhizal fungal activity (Fahey et al. 2022). AMF predominantly colonize plant roots in phosphorus-deficient environments, where they can satisfy over 80% of the host’s phosphorus requirements through two primary mechanisms: (1) recruiting phosphatase-producing bacteria to the rhizosphere, thereby enhancing phosphorus mineralization, and (2) releasing organic acids that solubilize phosphorus bound in iron and aluminum oxides, consequently increasing bioavailable phosphorus concentrations in the root zone (Zhang et al. 2018; Liu et al. 2021). While phosphorus acquisition represents their primary function, AMF can also contribute up to 25% of the host’s nitrogen demand in nitrogen-limited ecosystems (Marschner and Dell 1994). In contrast, EMF play a pivotal role in nitrogen-constrained forest ecosystems through dual mechanisms: symbiotic bacteria associated with EMF facilitate organic nitrogen decomposition, while EMF themselves secrete proteolytic enzymes to enhance nitrogen uptake by host roots (Wang et al. 2020; Rivera Pérez et al. 2022). These fungal groups exhibit distinct nutrient acquisition strategies: EMF can directly access organic matter through the secretion of oxidative enzymes for lignin degradation, whereas AMF, lacking comprehensive enzymatic capabilities, primarily depend on saprotrophic microorganisms for the conversion of organic nutrients into inorganic forms or the absorption of microbially-derived simple nutrients (Read and Perez-Moreno 2003; Talbot et al. 2015; Cao et al. 2022). The symbiotic relationship between these mycorrhizal fungi and their host plants is maintained through a reciprocal nutrient exchange, with plants typically allocating approximately 20% of their photosynthetic products to support fungal growth and maintenance (Jiang et al. 2017; Luginbuehl et al. 2017).

Given the crucial ecological functions of mycorrhizal fungi, investigations into their community structures have garnered substantial attention in soil mycological research. These community structures are influenced by environmental factors through both direct and indirect mechanisms. Empirical studies have demonstrated significant environmental influences: Shao et al. (2023) revealed that soil phosphorus availability and moisture content significantly affect the richness and composition of AMF communities in agricultural ecosystems, while Song et al. (2019) established strong correlations between AMF diversity and multiple soil parameters, including total nitrogen, available phosphorus, sucrase activity, soil organic matter, and urease activity. Similarly, Erlandson et al. (2016) identified soil moisture, pH, and organic matter content as primary determinants of EMF community dynamics in temperate rhizosphere soils. Host plant characteristics represent another critical factor shaping mycorrhizal fungal communities. Chen et al. (2020) demonstrated that host plant species accounted for 11.7% of the variation in AMF community structure in central United States forest ecosystems, though no significant host effect was observed for EMF communities in this context. In contrast, Carriconde et al. (2019) reported pronounced host-specific effects on EMF community structures in tropical rainforest ecosystems.

Mountain ecosystems characterized by significant altitudinal gradients, which induce marked variations in soil properties and plant communities, offer exceptional natural laboratories for exploring the influence of external factors on mycorrhizal fungi (Körner 2007; Afshana et al. 2020). However, the inherent altitudinal and geographical variability in mountain ecosystems complicates the identification of consistent patterns in mycorrhizal community structures. For instance, Zhao et al. (2022) reported a negative correlation between EMF richness and altitude in Qinling Mountain pine forests, primarily mediated by soil pH variations. In contrast, Rasmussen et al. (2022) found that both altitude and vegetation jointly influenced AMF community structures in Greenland. Climatic factors, which vary significantly along altitudinal gradients, also play a crucial role in shaping mycorrhizal fungal communities (Hiiesalu et al. 2023). The regional variability in climate change impacts further adds to this complexity. Despite the intricate interplay of external factors influencing mycorrhizal fungi in mountain ecosystems, research on altitudinal gradients and their underlying mechanisms remains crucial for advancing our understanding of mycorrhizal fungal community dynamics. This line of investigation continues to be of paramount importance in ecological and mycological research.

Community assembly mechanisms constitute a fundamental aspect in understanding fungal community structure dynamics. Two predominant theoretical frameworks currently guide this research domain. The niche theory emphasizes that deterministic processes—including species-specific traits, interspecific interactions, and environmental filtering—play a primary role in shaping community assembly (Zheng et al. 2021; Hu et al. 2023). Conversely, the neutral theory maintains that stochastic processes—encompassing birth-death dynamics, migration events, and dispersal limitations—serve as the principal determinants of community structure formation (Jiao et al. 2020; Hu et al. 2023). Advancing this theoretical framework, Ning et al. (2020) developed the integrated Community Assembly Mechanisms Prediction (iCAMP) method, an innovative phylogenetic bin-based null model analysis that enables quantitative assessment of community assembly mechanisms through the examination of pairwise phylogenetic turnover within microbial communities. This methodological approach classifies ecological processes into five distinct categories: heterogeneous selection and homogeneous selection (representing deterministic processes), along with dispersal limitation, homogenizing dispersal, and drift (characterizing stochastic processes). The application of iCAMP has yielded significant insights into fungal community assembly patterns. Li et al. (2023) employed this method to analyze soil fungal community assembly in Robiniapseudoacacia forests, identifying ecological drift as the predominant assembly process. Similarly, Wang et al. (2022c) utilized iCAMP to investigate soil fungal community assembly patterns in the southern Tibetan Plateau, demonstrating the predominance of stochastic processes in this high-altitude ecosystem. Despite these methodological advancements and empirical findings, the application of iCAMP to elucidate mycorrhizal fungal community assembly mechanisms, particularly within the context of mountain forest ecosystems, remains relatively unexplored. Further investigations employing this approach could provide critical insights into the ecological processes governing these ecologically vital fungal communities, potentially advancing our understanding of mountain ecosystem functioning and resilience.

Ecosystem multifunctionality, a pivotal ecological metric, quantifies the ability of an ecosystem to sustain multiple functions concurrently, such as nutrient cycling and organic matter decomposition (Wang et al. 2022b; Scherzinger et al. 2024). This multifunctionality is shaped by a multitude of factors, with microbial communities playing a central role in regulating ecological processes across spatial and temporal scales (Guo et al. 2021). Empirical evidence underscores that microbial diversity and soil properties—such as moisture, carbon, and nitrogen content—are critical determinants of ecosystem multifunctionality (Delgado-Baquerizo et al. 2017; Wang et al. 2022b). Additionally, vegetation dynamics and climatic conditions exert significant influence, further modulating this multifaceted metric (Giling et al. 2019; Wang et al. 2022b; Chen et al. 2023). Mountain forests, characterized by pronounced environmental heterogeneity, offer an ideal natural laboratory for investigating these drivers. For instance, research conducted in the Helan Mountain region has elucidated the complex interplay among microbial communities, topography, and climate in shaping ecosystem multifunctionality (Yang et al. 2023b), highlighting the intricate relationships that underpin this ecological phenomenon.

Wuyi Mountain is a globally significant biodiversity hotspot and represents one of the most intact mid-subtropical mountain ecosystems in eastern China. Its pronounced altitudinal gradients, favorable climate, and exceptional biodiversity establish it as an ideal natural laboratory for advanced microbiological research (Yang et al. 2023a). We conducted an extensive survey of both biotic and abiotic factors across five distinct altitudinal zones, with AMF and EMF communities analyzed using high-throughput Illumina sequencing technology. This study seeks to address three key questions: (1) How do altitudinal gradients influence the community structure and composition of AMF and EMF in Wuyi Mountain? (2) Do AMF and EMF communities demonstrate distinct assembly mechanisms? (3) What are the relative contributions of environmental factors in driving ecosystem multifunctionality along the altitudinal gradient?

## ﻿Material and methods

### ﻿Study sites and sampling

Our study was conducted in the Wuyi Mountain National Nature Reserve (27°73′–27°86′N, 117°69′–117°78′E), located in the northwestern region of Fujian Province, China (Suppl. material 1: table S1). The reserve is characterized by a typical subtropical monsoon climate, marked by a pronounced altitudinal gradient and notable climatic variations. The mean annual temperature (MAT) ranges from 9.14°C to 14.65°C, and the mean annual precipitation (MAP) varies between 1961.00 mm and 2308.00 mm. Spanning an area of 290 square kilometers, the reserve is home to extensive native vegetation (Li et al. 2021) (Suppl. material 1: table S2). Five study sites were selected at elevations of 675 m, 1227 m, 1762 m, 1852 m, and 2157 m, representing distinct vegetation types: evergreen broad-leaved forest (EBF), coniferous and broad-leaved mixed forest (CBMF), coniferous forest (CF), subalpine dwarf forest (SDF), and alpine meadow (ALM) (Suppl. material 1: table S1). The dominant species in each respective forest type include *Castanopsiseyrei*, *Syzygiumaustrosinense*, and *Cyclobalanopsismyrsinifolia* in the EBF; *Pinustaiwanensis*, *Rhododendronovatum*, *Euryamuricata*, and *Cyclobalanopsismultinervis* in the CBMF; *Pinustaiwanensis* and *Tsugachinensis* in the CF; *Euryasaxicola*, *Symplocoslucida*, *Yushaniahirticaulis*, *Deyeuxiapyramidalis*, and *Cyperusmicroiria* in the SDF; and *Miscanthussinensis*, *Aniselytrontreutleri*, and *Carexolivacea* in the ALM.

At each altitudinal level, three replicate study plots (20 m × 20 m) were systematically established. Each plot was subsequently divided into 16 contiguous quadrats (5 m × 5 m) through grid-based partitioning, with division boundaries precisely aligned at 5-meter intervals along both the × and y axes. A comprehensive tree survey was carried out within each quadrat. Three quadrats were randomly selected, and within each, five soil samples were collected from a depth of 0–15 cm in an S-shaped pattern. These samples were then homogenized and mixed to form a single composite sample. The samples were placed in sterile polyethylene bags, stored in an icebox, and transported to the laboratory within one day. Each composite sample was split into two subsamples: one for analysis of soil physicochemical properties and enzyme activities, and the other for storage at -80°C for total DNA extraction. The geographic coordinates of each sampling point were recorded using a global positioning system. Leaf Area Index (LAI), Enhanced Vegetation Index (EVI), and Normalized Difference Vegetation Index (NDVI) data were obtained from the LAADS DAAC (https://ladsweb.modaps.eosdis.nasa.gov) and extracted by latitude and longitude using the Environment for Visualizing Images software (Suppl. material 1: table S3).

### ﻿Soil physical and chemical properties and enzyme activities

The concentrations of soil total carbon (TC) and total nitrogen (TN) were analyzed using an elemental analyzer (FLASH SMART; Shanghai, China). Total phosphorus (TP) concentration was determined by alkali fusion molybdenum-antimony resistance spectrophotometry (Duan et al. 2023). Ammonium nitrogen (NH_4_^+^-N) and nitrate nitrogen (NO_3_^−^-N) concentrations were measured using indophenol blue colorimetry and phenol-disulfonic acid colorimetry, respectively (Zhao et al. 2022). Soil organic matter content was quantified through potassium dichromate oxidation spectrophotometry (Cen et al. 2021). Microbial biomass carbon (MBC), nitrogen (MBN), and phosphorus (MBP) were determined using the chloroform fumigation extraction method (Xu et al. 2021). Soil pH was measured with a pH meter (PB-10, Shanghai, China) in a 2.5:1 (water:soil) suspension, and electrical conductivity (EC) was measured with a conductivity meter (DDS-307A, Shanghai, China) in a 1:1.5 (water:soil) suspension. Soil moisture content was determined by the oven drying method (O’Kelly 2004).

The activities of soil enzymes were assessed as follows: urease activity was measured using indigo colorimetry, acid phosphatase activity by 2,3,5-triphenyl tetrazolium chloride colorimetry, dehydrogenase activity by triphenyltetrazolium chloride colorimetry, and leucine aminopeptidase activity by p-nitroaniline colorimetry. Cellulase and sucrase activities were determined using 3,5-dinitrosalicylic acid colorimetry, while β-1,4-glucosidase, β-1,4-N-acetylglucosaminidase, and β-xylosidase activities were quantified using the p-nitrophenol method (Mao and Jiang 2021; Xie et al. 2021; Boteva et al. 2022; Wang et al. 2022a; Lu et al. 2023; Qu et al. 2023).

### ﻿High-throughput sequencing

Total DNA was extracted from soil samples using the Fast DNA™ Spin Kit for Soil (MP Biomedicals, California, USA) following the manufacturer’s protocol. The concentration and quality of the extracted DNA were evaluated by 1% agarose gel electrophoresis. For fungal community analysis, the ITS1 region of the rRNA gene was amplified using the primer pair ITS1F (CTTGGTCATTTAGAGGAAGTAA) and ITS2R (GCTGCGTTCTTCATCGATGC) (Bell et al. 2020). Polymerase chain reaction (PCR) was conducted in a 20-μl reaction mixture containing 10 μl of 2× Pro Taq Master Mix, 0.8 μl each of forward and reverse primers (5 μM), 50 ng of template DNA, and ddH_2_O to adjust the final volume to 20 μl. The PCR cycling protocol included an initial denaturation at 95°C for 3 minutes, followed by 35 cycles of denaturation at 95°C for 30 seconds, annealing at 55°C for 30 seconds, and extension at 72°C for 45 seconds, with a final elongation step at 72°C for 10 minutes and a hold at 10°C.

For AMF, the V4–V5 hypervariable regions of the 18S rRNA gene were amplified using a nested PCR approach. The first round of PCR utilized the primer pair AML1F (ATCAACTTTCGATGGTAGGATAGA) and AML2R (GAACCCAAACACTTTGGTTTCC) (Yang et al. 2021) in a 20-μl reaction system with 2× Pro Taq Master Mix. The cycling conditions consisted of an initial denaturation at 95°C for 3 minutes, followed by 32 cycles of 95°C for 30 seconds, 55°C for 30 seconds, and 72°C for 45 seconds, with a final extension at 72°C for 10 minutes. The second round of PCR employed the primers AMV4-5NF (AAGCTCGTAGTTGAATTTCG) and AMDGR (CCCAACTATCCCTATTAATCAT) (Yang et al. 2021), with the following conditions: 95°C for 3 minutes, followed by 25 cycles of 95°C for 30 seconds, 55°C for 30 seconds, and 72°C for 45 seconds, and a final elongation at 72°C for 10 minutes. To prevent contamination, a negative control (ddH_2_O) was included during DNA extraction and PCR procedures.

The PCR products were initially visualized on a 2% agarose gel and subsequently quantified using the QuantiFluor™-ST Blue fluorescence quantification system (Promega, Madison, USA). Based on the quantification results, the PCR products were pooled in equimolar ratios according to the sequencing requirements for each sample. Sequencing libraries were prepared using the TruSeq™ DNA Sample Prep Kit (Sangon Biotech, Shanghai, China) and sequenced on the Illumina HiSeq 2500 platform (Illumina, Inc., San Diego, USA) at Majorbio Co. (Shanghai, China).

### ﻿Bioinformatics workﬂow

Paired-end reads generated on the Illumina platform were first merged using FLASH version 1.2.11 (Magoc and Salzberg 2011) based on overlap relationships. Adapters and low-quality sequences were then trimmed using Trimmomatic version 0.33, and chimeric sequences were detected and removed with UCHIME version 8.1 (Edgar et al. 2011; Bolger et al. 2014). The resulting high-quality, non-chimeric sequences were clustered into operational taxonomic units (OTUs) at a 97% similarity threshold using USEARCH version 11 (Edgar 2013). Fungal taxonomic classification was performed by aligning sequences against the UNITE reference database (version 7.0; https://unite.ut.ee/) (Nilsson et al. 2019). AMF were taxonomically assigned by querying representative sequences against the MaarjAM database (Öpik et al. 2010), while EMF were identified by cross-referencing the FungalTraits database (version 1.2) for lineages and exploration types associated with ectomycorrhizal lifestyles (Põlme et al. 2020). OTU-level taxonomic assignments were conducted using the RDP Classifier version 2.13 with an 80% confidence threshold (Wang et al. 2007). To account for uneven sequencing depths across samples, sequences were rarefied to the minimum depth using the “sub.sample” command in MOTHUR (version 1.46.0) (Schloss et al. 2009).

### ﻿Statistical analysis

Most data analyses in this study were performed using R (version 4.2.2). The α-diversity and phylogenetic diversity of mycorrhizal fungi were calculated using the “vegan” (version 2.7-0) and “picante” (version 1.8.2) R packages (Kembel et al. 2010). Fungal community differences were assessed through principal coordinates analysis (PCoA) and permutational multivariate analysis of variance (PERMANOVA). The influence of environmental factors on vertical community composition was evaluated using the “ecodist” (version 2.1.3) package (Goslee and Urban 2007). To explore the effects of altitude on fungal community composition, β-diversity was partitioned into species turnover and richness differences using the “adespatial” (version 0.3-24) package (Dray and Dufour 2007). Additionally, the “tidyverse” (version 2.0.0) package (Wickham et al. 2019) was employed to analyze the species composition of AMF and EMF in relation to altitudinal gradients.

Fungal community assembly mechanisms were elucidated by quantitatively evaluating the relative contributions of distinct ecological processes using the “iCAMP” package (version 1.5.12). In this framework, a βNRI value < -1.96 indicates homogeneous selection, while a βNRI value > 1.96 signifies heterogeneous selection. When |βNRI| ≤ 1.96 and the Raup-Crick (RC) value < -0.95, the process is identified as homogeneous dispersal. Conversely, |βNRI| ≤ 1.96 and RC > 0.95 suggest dispersal limitation. Finally, |βNRI| ≤ 1.96 and |RC| ≤ 0.95 are indicative of ecological drift (Ning et al. 2020). To identify potential keystone taxa within fungal communities, the specificity and occupancy rates of each OTU across altitudinal gradients were calculated and visualized (Gweon et al. 2021). The impact of environmental factors on the topological properties of mycorrhizal fungal networks was assessed using the “corr.test” function from the “psych” R package (version 2.4.6.26).

To investigate interactions within mycorrhizal fungal communities, co-occurrence networks were constructed using the weighted gene co-expression network analysis (WGCNA) framework in R (Langfelder and Horvath 2012). OTUs were represented as network nodes, and statistically significant associations between taxa were depicted as edges. To minimize false positives, multiple hypothesis testing corrections were applied using the “multtest” package (version 2.54.0) (Benjamini et al. 2006), with a significance threshold of α = 0.01. Indirect correlations were removed using network topology disentanglement algorithms (Feizi et al. 2015), retaining only robust pairwise relationships that met dual criteria: an absolute Spearman’s ρ > 0.6 and an adjusted P-value < 0.01 (Ma et al. 2015; Qian et al. 2019). Final network visualizations were produced using the Gephi platform (version 0.9.3; https://gephi.org), with node spatial arrangements optimized through force-directed algorithms (Mathieu et al. 2014).

Functional diversity metrics—including functional divergence and functional evenness—were calculated for mycorrhizal fungi based on community traits (e.g., richness, Shannon diversity, phylogenetic diversity, mean pairwise distance, network modularity, network density, node count, and edge count) and environmental preferences (e.g., pH, soil moisture content, MAT, and EC) using the “FD” package (version 1.0-12.3) (Escalas et al. 2019). Statistical significance across groups was assessed using one-way ANOVA and the aovMcomper function from the “EasyStat” package (version 0.1.0) when data met assumptions of normality and homogeneity of variance. Otherwise, the Kruskal-Wallis test and the KwWlx2 function were employed to evaluate overall and pairwise differences.

The influence of environmental factors on mycorrhizal fungal diversity was assessed using the Mantel test, implemented with the “linkET” package (version 0.0.7.3) (Liu et al. 2022). Following the approach of Yang et al. (2023b), TC, TN, TP, NO_3_^−^-N, NH_4_^+^-N, MBC, MBN, MBP, sucrase, urease, and leucine aminopeptidase were selected as proxies for ecological functions, representing soil nutrient storage, nutrient cycling, productivity, and fertility. Ecosystem multifunctionality was quantified using the mean value method, calculated as the average of Z-normalized individual function values. Structural equation modeling (SEM) and path coefficient analysis were conducted to evaluate the effects of altitude, climate, soil properties, and fungal communities on ecosystem multifunctionality, utilizing the “piecewiseSEM” package (version 2.3.0) (Liu et al. 2022). The relative importance of environmental factors to ecosystem multifunctionality was determined using a random forest model, implemented with the “rfPermute” package (version 2.5.2) (Zhang et al. 2022b). Non-linear regression was employed to model the relationships between mycorrhizal fungal diversity, altitude, and ecosystem multifunctionality, with the coefficient of determination (R²) and P-values of the fitted curves calculated using the lm function (Milici et al. 2016; Chen et al. 2022; Peng et al. 2023).

## ﻿Results

### ﻿Mycorrhizal fungal community composition

AMF were classified into five distinct orders, with *Glomerales* exhibiting the highest relative abundance, ranging from 79.14% to 97.70%. This order was most abundant in EBF at lower altitudes and least abundant in SDF at intermediate altitudes. *Diversisporales* followed, with a relative abundance ranging from 1.79% to 19.12% (Suppl. material 1: fig. S1A). In contrast, EMF were primarily divided into two phyla: *Basidiomycota*, which consistently exceeded 96% relative abundance across all altitudinal gradients, and *Ascomycota*, which showed a relative abundance ranging from 1.39% to 3.29% (Suppl. material 1: fig. S1B). At the genus level, the AMF community comprised 15 genera, while the EMF community included 62 genera. *Glomus* was the dominant genus for AMF across all altitudes. In contrast, the dominant EMF genera varied with altitude: *Russula* was prevalent in CBMF and SDF at intermediate altitudes; *Sebacina* dominated in EBF at the lowest altitudes; and *Entoloma* was most abundant in ALM at the highest altitudes (Suppl. material 1: fig. S1C, D).

### ﻿Mycorrhizal fungal diversity

**Figure 1.**
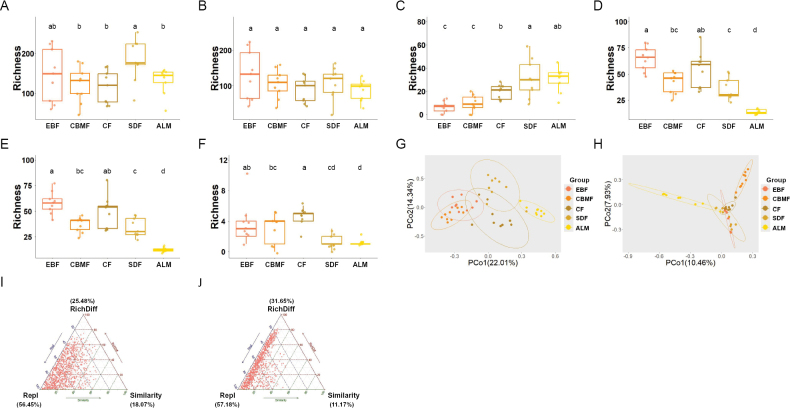
Altitudinal variation in mycorrhizal fungal richness and community differentiation. Richness of various taxa of mycorrhizal fungi including Arbuscular (**A**), *Glomerales* (**B**), *Diversisporales* (**C**), ectomycorrhizal fungi (**D**), *Basidiomycota* (**E**), and *Ascomycota* (**F**) across five altitudinal gradient zones. Community differences among arbuscular and ectomycorrhizal fungi are shown in **G** and **H**, respectively, while **I** and **J** depict the decomposition of β-diversity within these fungal communities. Repl: replacement; RichDiff: richness difference.

AMF richness peaked significantly in SDF at intermediate altitudes (Fig. 1A). Within this community, *Glomerales*, the dominant order in Wuyi Mountain, exhibited no significant altitudinal variation in richness (Fig. 1B). In contrast, *Diversisporales*, the second most abundant order, showed considerable variability in richness across the studied altitudinal gradients (Fig. 1C). Similarly, the EMF community displayed significant variations in richness, with the highest values observed in EBF at the lowest altitude and the lowest in ALM at the highest altitude (Fig. 1D). Richness within the *Basidiomycota* and *Ascomycota* phyla of the ectomycorrhizal community also exhibited significant altitudinal variations (Fig. 1E, F). PCoA revealed distinct community structures for both AMF and EMF across different altitudes (Fig. 1G, H). This differentiation was further supported by PERMANOVA, which confirmed significant altitudinal effects on community composition for both fungal types (Fig. 1G, H; Suppl. material 1: table S4). Environmental factor analysis identified strong correlations between fungal community variations and a range of soil and climatic parameters, including soil moisture, enzymatic activities, microbial biomass, nutrient concentrations, and vegetation indices (Suppl. material 1: table S5). Decomposition of β-diversity indicated that species replacement was the primary driver of community differences in both AMF and EMF, accounting for 56.45% and 57.18% of the variation, respectively, with additional contributions from richness differences and similarity (Fig. 1I, J).

AMF richness showed no significant correlation with altitude (P = 0.601) (Suppl. material 1: fig. S2A). *Glomerales* exhibited consistent richness across altitudinal gradients (P = 0.218), whereas the richness of *Diversisporales* increased significantly with altitude (P < 0.001, R² = 0.481) (Suppl. material 1: fig. S2B, C). In contrast, EMF communities, including both *Ascomycota* and *Basidiomycota*, displayed significant altitudinal fluctuations in richness, characterized by an initial decline, followed by an increase, and a subsequent decrease (P < 0.05, R² > 0.230) (Suppl. material 1: fig. S2D–F). We further investigated altitudinal trends in phylogenetic and functional diversity among mycorrhizal fungi. AMF phylogenetic diversity and functional evenness showed no significant vertical variation (P > 0.560, R² = 0.048) (Suppl. material 1: fig. S2G, I). However, AMF functional divergence exhibited an inverted S-shaped pattern with increasing altitude (P < 0.001, R² = 0.566) (Suppl. material 1: fig. S2H). EMF phylogenetic diversity followed a similar fluctuating pattern with altitude, while functional evenness was inversely correlated, and functional divergence remained stable (P = 0.515, R² = 0.001) (Suppl. material 1: fig. S2G–L).

**Figure 2.**
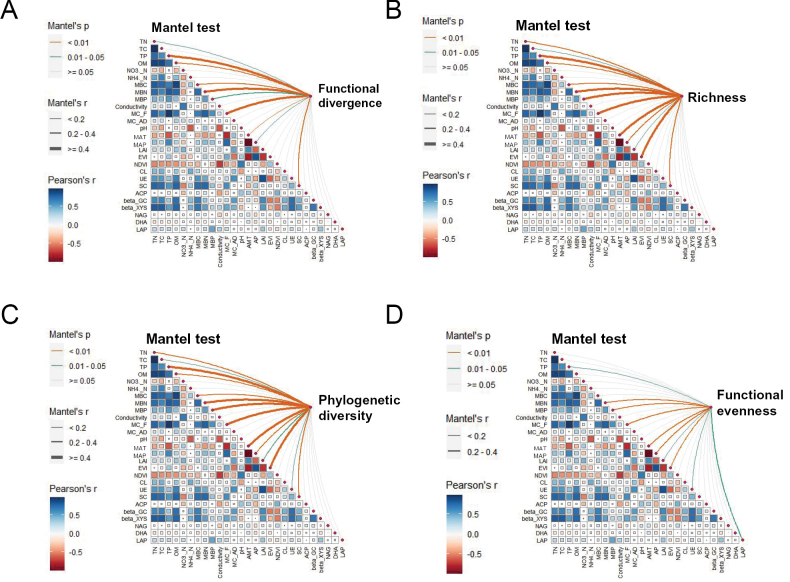
Influence of environmental variables on mycorrhizal fungal diversity and function. Environmental factors impact the functional diversity of arbuscular mycorrhizal fungi (**A**), richness (**B**), phylogenetic diversity (**C**), and functional evenness (**D**) of ectomycorrhizal fungi. TC: total carbon; TN: total nitrogen; TP: total phosphorus; OM: organic matter; MBC: microbial biomass carbon; MBN: microbial biomass nitrogen; MBP: microbial biomass phosphorus; NO3._N: nitrate nitrogen; NH4._N: ammonium nitrogen; EC: electric conductivity; MC_F: fresh soil moisture content; MC_AD: air-dried soil moisture content; MAT: mean annual temperature; MAP: mean annual precipitation; CL: cellulase; UE: urease; SC: sucrase; ACP: acid phosphatase; β-GC: β-1,4-glucosidase; DHA: dehydrogenase; NAG: β-1,4-N-acetylglucosaminidase; β-XYS: β-xylosidase; LAP: Leucine aminopeptidase.

Further analyses examined the influence of altitude on mycorrhizal fungal community diversity and its associations with environmental variables. The richness of *Diversisporales* showed significant correlations with soil moisture content, MAT, precipitation, and the EVI (P < 0.05) (Suppl. material 1: fig. S3A). AMF functional divergence was significantly linked to TN, TP, organic matter, microbial biomass, soil moisture, climatic conditions, and sucrase activity (P < 0.05) (Fig. 2A). EMF richness, particularly within *Basidiomycota*, was significantly associated with TC, TN, TP, organic matter, microbial biomass, soil moisture, and climatic variables (P < 0.05) (Fig. 2B and Suppl. material 1: fig. S3B). *Ascomycota* richness was significantly related to MAT, precipitation, EVI, and dehydrogenase activity (P < 0.05) (Suppl. material 1: fig. S3C). EMF phylogenetic diversity was correlated with similar soil and climatic parameters, including urease activity (P < 0.05) (Fig. 2C). Finally, EMF functional evenness exhibited significant relationships with TP, MBN, MBP, soil moisture, climatic variables, urease, and leucine aminopeptidase (P < 0.05) (Fig. 2D).

### ﻿Mycorrhizal fungal community assembly mechanism

**Figure 3.**
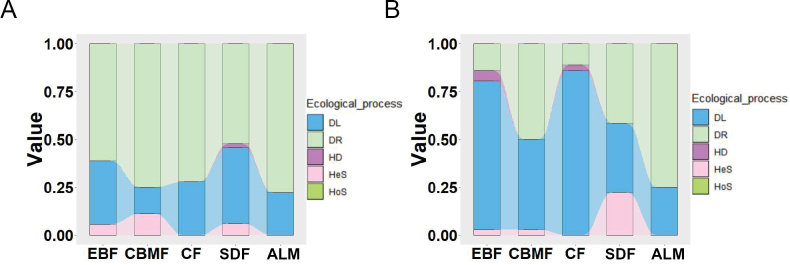
Dominance of ecological processes in mycorrhizal fungal community assembly. The relative importance of heterogeneous selection, homogeneous selection, dispersal limitation, homogenizing dispersal, and drift in shaping arbuscular (**A**) and ectomycorrhizal (**B**) fungal communities. HeS: heterogeneous selection; HoS: homogeneous selection; DL: dispersal limitation; HD: homogenizing dispersal; DR: drift.

The iCAMP analysis revealed that dispersal limitation and ecological drift were the primary drivers structuring the assembly of the two distinct mycorrhizal fungal communities, collectively accounting for over 86.50% of the observed assembly processes (Suppl. material 1: fig. S4). In contrast, the contributions of the other three ecological processes were relatively minor, representing less than 13.45% of the assembly dynamics (Suppl. material 1: fig. S4). The assembly of mycorrhizal fungi was further influenced by both altitude and fungal type. Specifically, ecological drift dominated the community assembly of AMF, exhibiting a complex pattern of increase, decrease, and subsequent increase with altitude (Fig. 3A). In contrast, dispersal limitation was the primary factor shaping the assembly of EMF, displaying a pattern inverse to that of AMF (Fig. 3B). Homogenizing dispersal was observed exclusively at intermediate altitudes in AMF communities, while it occurred at both the lowest and intermediate altitudes in EMF communities (Fig. 3A, B). Heterogeneous selection was detected in both fungal types at the same altitude, though its influence varied between the communities (Fig. 3A, B).

### ﻿Potential keystone fungal taxa

SPEC-OCCU plot analyses revealed that AMF and EMFOTUs across various altitudes did not cluster within specific x-axis columns, indicating a high diversity of occupancy characteristics at different elevations (Suppl. material 1: fig. S5). The study identified potential keystone taxa—defined as OTUs with specificity and occupancy values no less than 0.7—across five altitudinal stands, demarcated by dotted lines. As altitude increased, the distribution of potential keystone OTUs in AMF communities exhibited significant variation, representing 0.14, 0.33, 0.01, 0.27, and 0.28 of the community at each respective altitude (Suppl. material 1: fig. S5A–E). Similarly, the proportion of potential keystone OTUs within EMF communities varied, comprising 0.16, 0.65, 0.03, 0.56, and 0.62 from the lowest to the highest altitudes (Suppl. material 1: fig. S5F–J).

Within the AMF community, *Glomerales* were identified as potential keystone taxa across all forest belts. *Diversisporales* appeared at intermediate altitudes in CF and SDF, and at the highest altitudes in ALM, while *Archaeosporales* were exclusively observed in SDF at intermediate altitudes (Suppl. material 1: fig. S5A–E). For the EMF community, *Basidiomycota* and *Ascomycota* emerged as potential keystone taxa; *Basidiomycota* were found at all altitudes, while *Ascomycota* were specifically observed in the CBMF at intermediate altitudes and in ALM at the highest altitudes (Suppl. material 1: fig. S5F–J).

### ﻿Mycorrhizal fungal co-occurrence networks

**Figure 4.**
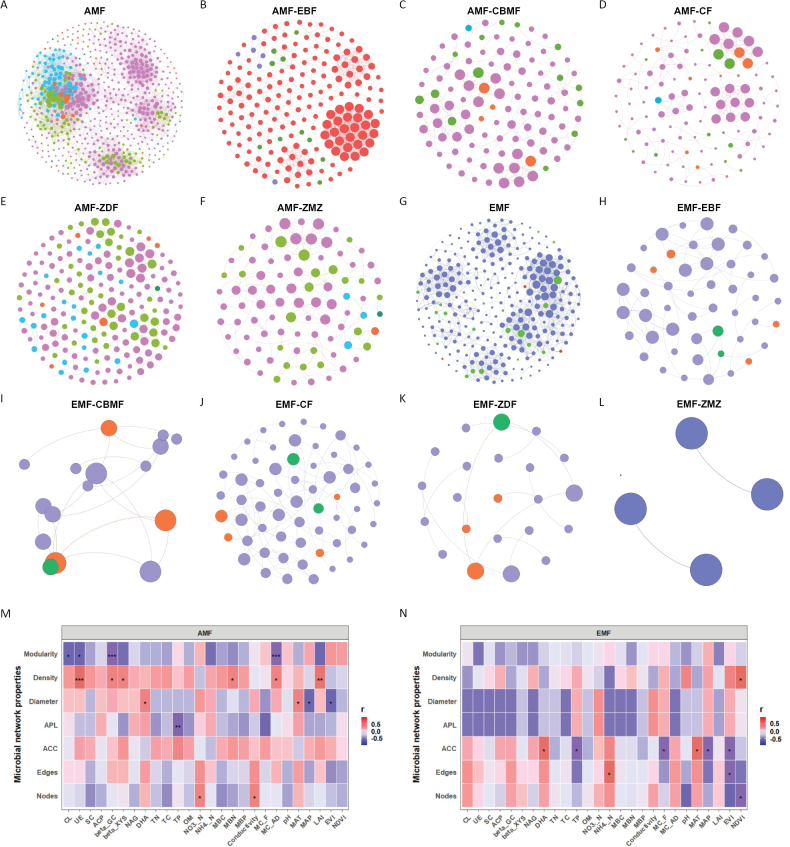
Co-occurrence networks of mycorrhizal fungal communities across different ecosystems. Total and specific community networks of arbuscular and ectomycorrhizal fungi in various forest types (**A–L**), with analysis of the relationship between network topological features and environmental factors for arbuscular (**M**) and ectomycorrhizal fungi (**N**). EBF: evergreen broad-leaved forest; CBMF: coniferous and broad-leaved mixed forest; CF: coniferous forest; SDF: subalpine dwarf forest; ALM: alpine meadow. ACC: average clustering coefficient; APL: average path length.

Co-occurrence networks for AMF and EMF consisted of 665 and 281 nodes, respectively, connected by 6,302 and 1,416 edges (Fig. 4A, G; Suppl. material 1: table S6). The AMF network exhibited greater complexity than the EMF network, although it displayed lower values for average clustering coefficient, average path length, diameter, density, and modularity (Fig. 4A, G; Suppl. material 1: table S6). Variations in network complexity were observed across altitudinal gradients. Specifically, the AMF network was most complex in EBF at the lowest altitude and least complex in ALM at the highest altitude. Conversely, the EMF network reached peak complexity in CF at intermediate altitudes and was least complex in ALM at the highest altitude (Fig. 4B–F, H–L; Suppl. material 1: table S6). Notably, no shared edges or nodes were observed between AMF and EMF networks across the five studied altitudes (Suppl. material 1: fig. S6). Topological features of these networks, including average clustering coefficient, path length, diameter, density, and modularity, exhibited significant altitudinal variations (Suppl. material 1: table S6).

Further analysis revealed relationships between network topology and environmental factors. In the AMF network, modularity was significantly associated with cellulase, urease, acid phosphatase, and soil moisture content. Network density showed strong correlations with urease, β-xylosidase, acid phosphatase, MBN, soil moisture content, and LAI. Diameter was significantly linked to dehydrogenase, MAT, precipitation, and EVI. Additionally, average path length correlated with TP, while nodes demonstrated significant associations with NO_3_^−^-N and EC (Fig. 4M). In the EMF network, density was significantly correlated with NDVI, and the average clustering coefficient showed significant relationships with dehydrogenase, TP, soil moisture content, MAT, precipitation, and EVI. Significant associations were also observed between edges and NH_4_^+^-N, EVI, while nodes were notably related to NDVI (Fig. 4N).

### ﻿Ecosystem multifunctionality

**Figure 5.**
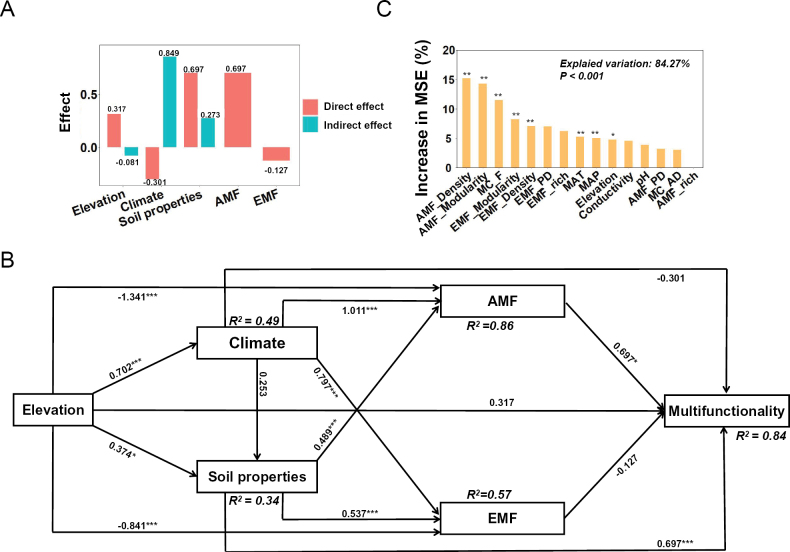
The relationships between ecosystem multifunctionality and environmental factors across altitudinal gradients. **A, B** direct and indirect effects of environmental factors on multifunctionality; **C** relative importance of individual environmental factors. EBF: evergreen broad-leaved forest; CBMF: coniferous and broad-leaved mixed forest; CF: coniferous forest; SDF: subalpine dwarf forest; ALM: alpine meadow; MC_F: fresh soil moisture content; MC_AD: air-dried soil moisture content; MAT: mean annual temperature; MAP: mean annual precipitation; AMF_PD; arbuscular mycorrhizal fungal phylogenetic diversity; AMF_rich: arbuscular mycorrhizal fungal richness; AMF_Modularity: arbuscular mycorrhizal fungal network modularity; AMF_Density: arbuscular mycorrhizal fungal network density; EMF_PD; ectomycorrhizal fungal phylogenetic diversity; EMF_rich: ectomycorrhizal fungal richness; EMF_Modularity: ectomycorrhizal fungal network modularity; EMF_Density: ectomycorrhizal fungal network density.

Ecosystem multifunctionality demonstrated significant variation along altitudinal gradients, with the highest levels observed in the ALM at the highest elevation and the lowest levels in the CBMF at intermediate elevations. To elucidate the drivers of this variability, we employed a SEM and analyzed the associated path coefficients. The results revealed both direct and indirect effects of multiple factors on multifunctionality. Soil properties emerged as the most influential factor, exhibiting a strong direct effect (0.697) and a moderate indirect effect (0.273). AMF also showed a substantial direct effect (0.697) (Fig. 5A, B). Climatic factors ranked third in terms of overall influence, with a total effect of 0.548 (Fig. 5A, B). Altitude had a direct positive effect (0.317) on multifunctionality but a slight negative indirect effect (-0.081), resulting in a net total effect of 0.236, which exceeded the effect of EMF alone (-0.127) (Fig. 5A, B). Additionally, random forest analysis (explained variation: 84.27%, P < 0.001) identified network modularity, network density, MAT, MAP, and altitude as the primary determinants of multifunctionality (Fig. 5C).

## ﻿Discussion

The AMF community exhibited maximum species richness in the SDF, which can be attributed to the extensive colonization of herbaceous plant roots that are abundant in both SDF and adjacent ALM ecosystems of Wuyi Mountains. This pattern aligns with existing literature demonstrating that harsh environmental conditions at higher elevations negatively impact habitat quality (Vieira et al. 2019; Bueno et al. 2021; Guy et al. 2022; Liu et al. 2023b). Furthermore, our analysis revealed significantly reduced phylogenetic distances among AMF species in ALM, suggesting intensified competitive interactions resulting from limited nutrient availability and restricted spatial resources (Mammola et al. 2021; Xia et al. 2022; Schlechter et al. 2023), ultimately leading to lower community richness compared to SDF. Regarding *Diversisporales*, we observed a positive correlation between species richness and elevation, likely due to reduced competitive pressure from *Glomerales*, which showed decreasing abundance with increasing altitude (Wandrag et al. 2019). This altitudinal gradient creates more favorable conditions for *Diversisporales* to access and utilize available resources. The EMF community displayed a complex, nonlinear response to elevation, characterized by an initial decrease, subsequent increase, and final decline at higher altitudes. This pattern appears to be strongly influenced by extreme climatic conditions, including low temperatures, high wind exposure, and intense ultraviolet radiation (Bahram et al. 2012; Gong et al. 2022). Notably, EMF demonstrated preferential colonization of *Pinaceae* and *Fagaceae* tree species, which dominate the EBF and CF forests of Wuyi Mountain. This observation suggests that forest vegetation type exerts a more substantial influence on EMF richness than altitudinal factors alone (Štursová et al. 2020; Hicks Pries et al. 2023).

The heterogeneous environmental gradients across Wuyi Mountain’s altitudinal transect exert substantial influences on the vertical stratification patterns of both AMF and EMF communities. Our findings strongly support the metabolic theory of ecology, demonstrating that climatic variables, particularly MAT, serve as pivotal drivers of mycorrhizal diversity through their regulation of soil fertility parameters and microbial metabolic processes (Bahram et al. 2012). This temperature-dependent pattern aligns with previous reports by Körner (2007) on EMF diversity and Bueno et al. (2017) on AMF colonization dynamics, confirming the fundamental relationship between thermal regimes and mycorrhizal community structure. Furthermore, our results corroborate the established significance of MAP as a determinant of mycorrhizal diversity, consistent with contemporary findings (Guo et al. 2020; Yang et al. 2022; Ma et al. 2023). The edaphic environment, particularly the spatial distribution of TN, TP, organic matter content, and microbial biomass (carbon, nitrogen, phosphorus), emerges as another critical regulator of mycorrhizal community composition. These biogeochemical parameters, which exhibit marked altitudinal variation, directly influence fungal metabolic activities and community assembly processes (Gong et al. 2022). Lilleskov et al. (2011) illustrated how nitrogen deposition could shift EMF communities from those adapted to low-nitrogen environments to those that exploit available phosphorus in nitrogen-rich settings.

Furthermore, while soil pH serves as a crucial regulator of nutrient availability and ion exchange dynamics, our study revealed no direct correlation with mycorrhizal diversity. This absence of correlation may be attributed to the non-limiting nature of phosphorus metabolism for mycorrhizal fungi in Wuyi Mountain’s forest ecosystems, as indicated by the lack of significant relationships between fungal richness and nitrogen- or phosphorus-acquiring enzymatic activities. Notably, we identified a strong positive correlation between mycorrhizal diversity and carbon-cycling enzymatic activity, highlighting the pivotal role of soil enzymes in mediating the transformation of organic matter into bioavailable nutrients that support microbial metabolic processes (Cui et al. 2019). Moreover, our findings demonstrate that secondary environmental variables, including soil moisture content, EC, and vegetation indices, significantly influence microbial metabolism and extracellular enzymatic activities. These factors collectively contribute to the altitudinal variation in mycorrhizal diversity through their modulation of belowground ecological processes (Fairbanks et al. 2020; Tang et al. 2020; Sveen et al. 2021). This complex interplay of abiotic and biotic factors underscores the multifaceted nature of mycorrhizal community assembly along environmental gradients.

Our findings provide comprehensive insights into the complex dynamics of deterministic and stochastic processes governing AMF and EMF community assembly in Wuyi Mountain’s heterogeneous landscape. While deterministic factors contribute to community structuring, our results corroborate Zheng et al. (2021)’s findings that stochastic processes predominantly drive community assembly patterns. Among these stochastic elements, dispersal limitation emerges as a critical factor, imposing substantial constraints on microbial mobility and generating pronounced spatial heterogeneity across the mountain ecosystem (Zhou and Ning 2017). This phenomenon not only emphasizes the stochastic nature of microbial dispersal but also reveals the intricate spatial dynamics inherent in ecological community assembly. Although mycorrhizal fungal spores possess multiple dispersal vectors, including wind currents, animal-mediated transport, and specialized biological mechanisms (Davison et al. 2016; Vályi et al. 2016), their effective dispersal is significantly constrained by low atmospheric spore concentrations and limited long-range dispersal success (Kivlin et al. 2014). These limitations are particularly pronounced in Wuyi Mountain’s rugged topography, where altitudinal migration faces substantial physical barriers, creating distinct ecological niches that shape community structures. Furthermore, our study highlights the significance of ecological drift, conceptualized by Vellend (2010) as random fluctuations in community composition driven by fundamental demographic processes (birth, death, and reproduction), as a crucial mechanism in AMF community assembly. The topographic complexity of Wuyi Mountain amplifies these stochastic effects, making internal succession processes and random demographic events more influential than deterministic environmental filtering. This effect is particularly pronounced in small-scale communities, where priority effects can substantially increase the relative importance of drift (Wang et al. 2020), creating unique assembly patterns across different microhabitats.

Network analysis has emerged as a powerful tool for elucidating the intricate interaction patterns within microbial communities (de Vries et al. 2018). Through comprehensive construction and analysis of AMF and EMF network structures across altitudinal gradients, we have systematically characterized how ecological and environmental variables shape community interactions and structural organization. Our findings reveal a positive correlation between community diversity and network complexity, supporting the ecological hypothesis that diverse communities develop more sophisticated and resilient inter-taxa interaction networks (Manoeli et al. 2014). The network dynamics demonstrate significant responsiveness to environmental gradients, with vegetation composition, precipitation patterns, and soil nutrient availability showing strong associations with community structural changes along the elevational transect (Liu et al. 2023a). Notably, the most simplified network structures and weakest fungal interactions were observed at higher altitudes, where extreme environmental conditions prevail. This pattern reflects the constraining effects of intense ultraviolet radiation, low temperatures, and limited biological resources on community complexity. The scarcity of keystone taxa, which typically play pivotal roles in maintaining ecological stability and network integrity (Shi et al. 2016), likely exacerbates this structural simplification in high-altitude ecosystems.

Ecosystem multifunctionality serves as a critical integrative metric for assessing ecosystem functional potential, with microbial communities playing a central regulatory role (Luo et al. 2018; Sasaki et al. 2022). Our investigation in Wuyi Mountain reveals a complex, context-dependent relationship between mycorrhizal fungal diversity and ecosystem multifunctionality. Specifically, we observed a positive correlation between AMF diversity and multifunctionality, contrasting with negative correlations for EMF diversity. This divergence reflects the dualistic nature of external influences on ecosystem functioning, where the net effect depends on the balance between positive and negative interactions (Wang et al. 2021, 2022b). These findings are substantiated by a growing body of empirical evidence (Han et al. 2022; Li et al. 2022; Zhang et al. 2022b; Sun et al. 2023). The ALM ecosystem exhibited peak multifunctionality, consistent with elevated values across multiple ecological function indicators. This pattern can be attributed to two primary mechanisms: (1) the favorable soil physicochemical properties characteristic of humid climate regions, where meadow ecosystems facilitate humus accumulation and maintain higher soil fertility (Zhang et al. 2024); and (2) the compensatory response of ecosystems to severe climatic conditions, which necessitates enhanced multifunctionality to maintain habitat stability.

The contrasting relationships between AMF/EMF diversity and multifunctionality likely stem from fundamental differences in microbial life-history strategies and niche complementarity. These ecological distinctions mediate the functional impacts of each mycorrhizal group, resulting in distinct functional trade-offs (Yang et al. 2023b). Furthermore, our analysis identified soil properties as key determinants of multifunctionality in Wuyi Mountain, consistent with previous research (Wang et al. 2022b). Random forest analysis revealed that network modularity and density significantly contribute to multifunctionality, potentially through a stress-compensation mechanism where environmental pressures destabilize microbial networks, triggering multifunctionality responses to maintain habitat functionality (Hernandez et al. 2021). Soil moisture content emerged as another critical factor influencing multifunctionality, primarily through its regulation of soil carbon and nitrogen storage and cycling dynamics (Falloon et al. 2011; Zhang et al. 2022a). The significant correlation between soil moisture and mycorrhizal diversity suggests an indirect pathway for moisture effects on multifunctionality, highlighting the intricate interplay between biotic and abiotic components in shaping ecosystem functioning.

## ﻿Conclusion

In summary, our study combines high-throughput sequencing and bioinformatics to map the elevation-driven distribution patterns of mycorrhizal fungal communities in a mid-subtropical mountain ecosystem. By systematically analyzing altitudinal effects on fungal diversity and ecosystem functions, we uncover key ecological mechanisms shaping these communities. However, knowledge gaps remain regarding fungal adaptations across elevation gradients. Future research should prioritize genomic studies to identify genes with significant abundance shifts along altitudinal gradients, offering deeper insights into fungal ecology and microbial adaptation in changing environments.

## ﻿Additional information

### Adherence to national and international regulations

All the fungal strains used in this study have been legally obtained, respecting the Convention on Biological Diversity (Rio Convention).

### Author ORCIDs

Chunping Wu https://orcid.org/0009-0000-5888-5444

### Data availability

Data will be made available on request.

## Supplementary materials

Supplementary material 1 Supplementary tables and figures Data type docx Explanation note **table S1**: Geographical information of the five forest belts at five altitudes. **table S2**: Climate information of the five forest belts. **table S3**: Vegetation indexes of the five forest belts. **table S4**: PERMANOVA results displaying the effects of elevation on the community composition of the two mycorrhizal fungi. **table S5**: Effects of environmental factors on vertical community differences of arbuscular mycorrhizal fungi and ectomycorrhizal fungi. **table S6**: Topological characteristics of mycorrhizal fungal networks and corresponding random networks. **figure S1**: Species composition of arbuscular mycorrhizal fungi in order level and genus level at five elevations. **figure S2**: The relationship between mycorrhizal fungal diversity and elevation. **figure S3**: Effects of environmental factors on arbuscular mycorrhizal fungal and ectomycorrhizal fungial diversity. **figure S4**: Relative importance of different ecological processes in mycorrhizal fungal community assembly. **figure S5**: Distribution of the 500 most abundant operational taxonomic units (OTUs) in SPEC-OCCU plots. **figure S6**: The Venn diagrams display the sharing edges and points of mycorrhizal fungi at 5 elevations. https://binary.pensoft.net/file/1298752This dataset is made available under the Open Database License (http://opendatacommons.org/licenses/odbl/1.0/). The Open Database License (ODbL) is a license agreement intended to allow users to freely share, modify, and use this Dataset while maintaining this same freedom for others, provided that the original source and author(s) are credited.Taotao Wei, Huiguang Zhang, Shunfen Wang, Chunping Wu, Tieyao Tu, Yonglong Wang, Xin Qian
