# Supplementary material for: ﻿Divergent altitudinal patterns of arbuscular and ectomycorrhizal fungal communities in a mid-subtropical mountain ecosystem
Source: IMA Fungus. 2025 Apr 3;16:e140187. doi: 10.3897/imafungus.16.e140187 (PMC11986432; doi:10.3897/imafungus.16.e140187)
Supplement: Supplementary material 1 — Supplementary tables and figures [file imafungus-16-e140187-s001.docx]

**Divergent Altitudinal Patterns of Arbuscular and Ectomycorrhizal Fungal Communities in a Mid-Subtropical Mountain Ecosystem**

Taotao Wei^1, 2^, Huiguang Zhang^3^, Shunfen Wang^2^, Chunping Wu^2^, Tieyao Tu^4^*, Yonglong Wang^5^*, Xin Qian^1^*

1 College of Forestry, Fujian Agriculture and Forestry University, Fujian, China

2 College of Life Sciences, Fujian Agriculture and Forestry University, Fujian, China

3 Fujian Provincial Forestry Survey and Planning Institute, Fujian, China

4 South China Botanical Garden, Chinese Academy of Sciences, Guangdong, China

5 Baotou Teachers’ College, Baotou, 014030, China

**Corresponding author: Xin Qian; E-mail: qxxb2006@163.com

Tieyao Tu; E-mail: tutieyao@scbg.ac.cn

Yonglong Wang; E-mail: [wylongceltics@163.com](mailto:wylongceltics@163.com)

**Table S1:** Geographical information of the five forest belts at five altitudes.

| Name | Lon(°E) | Lat(°N) | Altitude(m) |
| --- | --- | --- | --- |
| EBF | 117.6910 | 27.7318 | 675.31 |
| CBMF | 117.7215 | 27.8228 | 1226.92 |
| CF | 117.7620 | 27.8466 | 1761.57 |
| SDF | 117.7629 | 27.8338 | 1851.88 |
| ALM | 117.7837 | 27.8609 | 2156.62 |

**Table S2:** Climate information of the five forest belts.

| Name | MAT(℃) | MAP(mm) |
| --- | --- | --- |
| EBF | 1961.0000 | 14.6542 |
| CBMF | 2049.0000 | 13.1458 |
| CF | 2197.0000 | 11.0042 |
| SDF | 2251.0000 | 10.3833 |
| ALM | 2308.0000 | 9.1375 |

MAT: mean annual temperature, MAP: mean annual precipitation.

**Table S3:** Vegetation indexes of the five forest belts.

| Name | LAI | EVI | NDVI |
| --- | --- | --- | --- |
| EBF | 5.8000 | 0.3314 | 0.8851 |
| CBMF | 0.5000 | 0.3999 | 0.918 |
| CF | 0.7000 | 0.3795 | 0.8835 |
| SDF | 0.5000 | 0.4199 | 0.8897 |
| ALM | 2.0000 | 0.4307 | 0.8952 |

LAI: Leaf Area Index, NDVI: Normalized Difference Vegetation Index, EVI: Enhanced Vegetation Index.

EBF: evergreen broad-leaved forest, CBMF: oniferous and broad-leaved mixed forest, CF: coniferous forest, SDF: subalpine dwarf forest, ALM: alpine meadow.

**Table S4:** PERMANOVA results displaying the effects of elevation on the community composition of the two mycorrhizal fungi.

|  |  | PERMANOVA |  |
| --- | --- | --- | --- |
|  |  | *R^2^* | *P* |
| AMF | Total community | 0.419 | 0.001 |
|  | EBF / CBMF | 0.211 | 0.001 |
|  | EBF / CF | 0.250 | 0.001 |
|  | EBF / SDF | 0.239 | 0.001 |
|  | EBF / ALM | 0.414 | 0.001 |
|  | CBMF / CF | 0.277 | 0.001 |
|  | CBMF / SDF | 0.327 | 0.001 |
|  | CBMF / ALM | 0.498 | 0.001 |
|  | CF / SDF | 0.254 | 0.001 |
|  | CF / ALM | 0.312 | 0.001 |
|  | SDF / ALM | 0.309 | 0.001 |
| EMF | Total community | 0.259 | 0.001 |
|  | EBF / CBMF | 0.190 | 0.001 |
|  | EBF / CF | 0.122 | 0.001 |
|  | EBF / SDF | 0.141 | 0.001 |
|  | EBF / ALM | 0.208 | 0.001 |
|  | CBMF / CF | 0.163 | 0.001 |
|  | CBMF / SDF | 0.191 | 0.001 |
|  | CBMF / ALM | 0.269 | 0.001 |
|  | CF / SDF | 0.130 | 0.001 |
|  | CF / ALM | 0.195 | 0.001 |
|  | SDF / ALM | 0.197 | 0.001 |

EBF: evergreen broad-leaved forest; CBMF: coniferous and broad-leaved mixed forest; CF: coniferous forest; SDF: subalpine dwarf forest; ALM: alpine meadow; Repl: replacement; RichDiff: richness difference; AMF: arbuscular mycorrhizal fungi; EMF: ectomycorrhizal fungi.

**Table S5:** Effects of environmental factors on vertical community differences of arbuscular mycorrhizal fungi and ectomycorrhizal fungi.

|  | AMF | | EMF | |
| --- | --- | --- | --- | --- |
| Factor | *R* | *P* | *R* | *P* |
| CL | -0.015 | 0.579 | 0.018 | 0.317 |
| UE | 0.211 | 0.001 | 0.231 | 0.001 |
| SC | 0.171 | 0.002 | 0.112 | 0.003 |
| ACP | 0.042 | 0.194 | 0.130 | 0.001 |
| β_GC | 0.104 | 0.017 | 0.157 | 0.001 |
| β_XYS | 0.156 | 0.003 | 0.174 | 0.001 |
| NAG | -0.033 | 0.744 | 0.016 | 0.302 |
| DHA | -0.051 | 0.881 | 0.020 | 0.279 |
| TN | 0.269 | 0.001 | 0.241 | 0.001 |
| TC | 0.243 | 0.001 | 0.233 | 0.001 |
| TP | 0.320 | 0.001 | 0.220 | 0.001 |
| OM | 0.260 | 0.001 | 0.225 | 0.001 |
| NO_3_^-^_N | 0.291 | 0.001 | 0.211 | 0.001 |
| NH_4_^+^_N | 0.067 | 0.106 | 0.155 | 0.001 |
| MBC | 0.184 | 0.002 | 0.143 | 0.001 |
| MBN | 0.241 | 0.001 | 0.173 | 0.001 |
| MBP | 0.024 | 0.321 | 0.035 | 0.162 |
| Electric conductivity | 0.206 | 0.001 | 0.209 | 0.001 |
| MC_F | 0.386 | 0.001 | 0.264 | 0.001 |
| MC_AD | 0.100 | 0.019 | 0.054 | 0.061 |
| pH | 0.074 | 0.082 | 0.191 | 0.001 |
| MAT | 0.525 | 0.001 | 0.333 | 0.001 |
| MAP | 0.532 | 0.001 | 0.332 | 0.001 |
| LAI | 0.225 | 0.001 | 0.243 | 0.001 |
| EVI | 0.368 | 0.001 | 0.328 | 0.001 |
| NDVI | 0.235 | 0.001 | 0.246 | 0.001 |

EBF: evergreen broad-leaved forest; CBMF: coniferous and broad-leaved mixed forest; CF: coniferous forest; SDF: subalpine dwarf forest; ALM: alpine meadow; Repl: replacement; RichDiff: richness difference; AMF: arbuscular mycorrhizal fungi; EMF: ectomycorrhizal fungi; TC: total carbon; TN: total nitrogen; TP: total phosphorus; OM: organic matter; MBC: microbial biomass carbon; MBN: microbial biomass nitrogen; MBP: microbial biomass phosphorus; NO_3_^-^_N: nitrate nitrogen; NH_4_^+^_N: ammonium nitrogen; MC_F: fresh soil moisture content; MC_AD: air-dried soil moisture content; MAT: mean annual temperature; MAP: mean annual precipitation; CL: cellulase; UE: urease; SC: sucrase; ACP: acid phosphatase; β-GC: β-1,4-glucosidase; DHA: dehydrogenase; NAG: β-1,4-N-acetylglucosaminidase; β-XYS: β-xylosidase; LAP: Leucine aminopeptidase.

**Table S6:** Topological characteristics of mycorrhizal fungal networks and corresponding random networks

| Fungal community | | Empirical network | | | | | | |
| --- | --- | --- | --- | --- | --- | --- | --- | --- |
| Group | | Nodes | Edges | ACC | APL | Diameter | Density | Modularity |
| Species | AMF | 665 | 6302 | 0.559 | 4.75 | 12 | 0.029 | 0.669 |
|  | EBF | 185 | 678 | 0.921 | 1.179 | 7 | 0.040 | 0.700 |
|  | CBMF | 106 | 135 | 0.819 | 1.232 | 4 | 0.024 | 0.919 |
|  | CF | 113 | 165 | 0.819 | 1.226 | 4 | 0.026 | 0.857 |
|  | SDF | 190 | 291 | 0.908 | 1.179 | 4 | 0.016 | 0.945 |
|  | ALM | 90 | 110 | 0.968 | 1.101 | 3 | 0.027 | 0.914 |
|  | EMF | 281 | 1416 | 0.560 | 5.461 | 13 | 0.036 | 0.726 |
|  | EBF | 49 | 51 | 1.000 | 1.000 | 1 | 0.043 | 0.088 |
|  | CBMF | 16 | 15 | 1.000 | 1.000 | 1 | 0.125 | 0.744 |
|  | CF | 60 | 67 | 1.000 | 1.000 | 1 | 0.038 | 0.913 |
|  | SDF | 22 | 12 | 0.750 | 1.071 | 2 | 0.056 | 0.875 |
|  | ALM | 4 | 2 | / | 1.000 | 1 | 0.333 | 0.500 |

EBF: evergreen broad-leaved forest; CBMF: coniferous and broad-leaved mixed forest; CF: coniferous forest; SDF: subalpine dwarf forest; ALM: alpine meadow; ACC: average clustering coefficient; APL: average path length; AMF: arbuscular mycorrhizal fungi; EMF: ectomycorrhizal fungi.


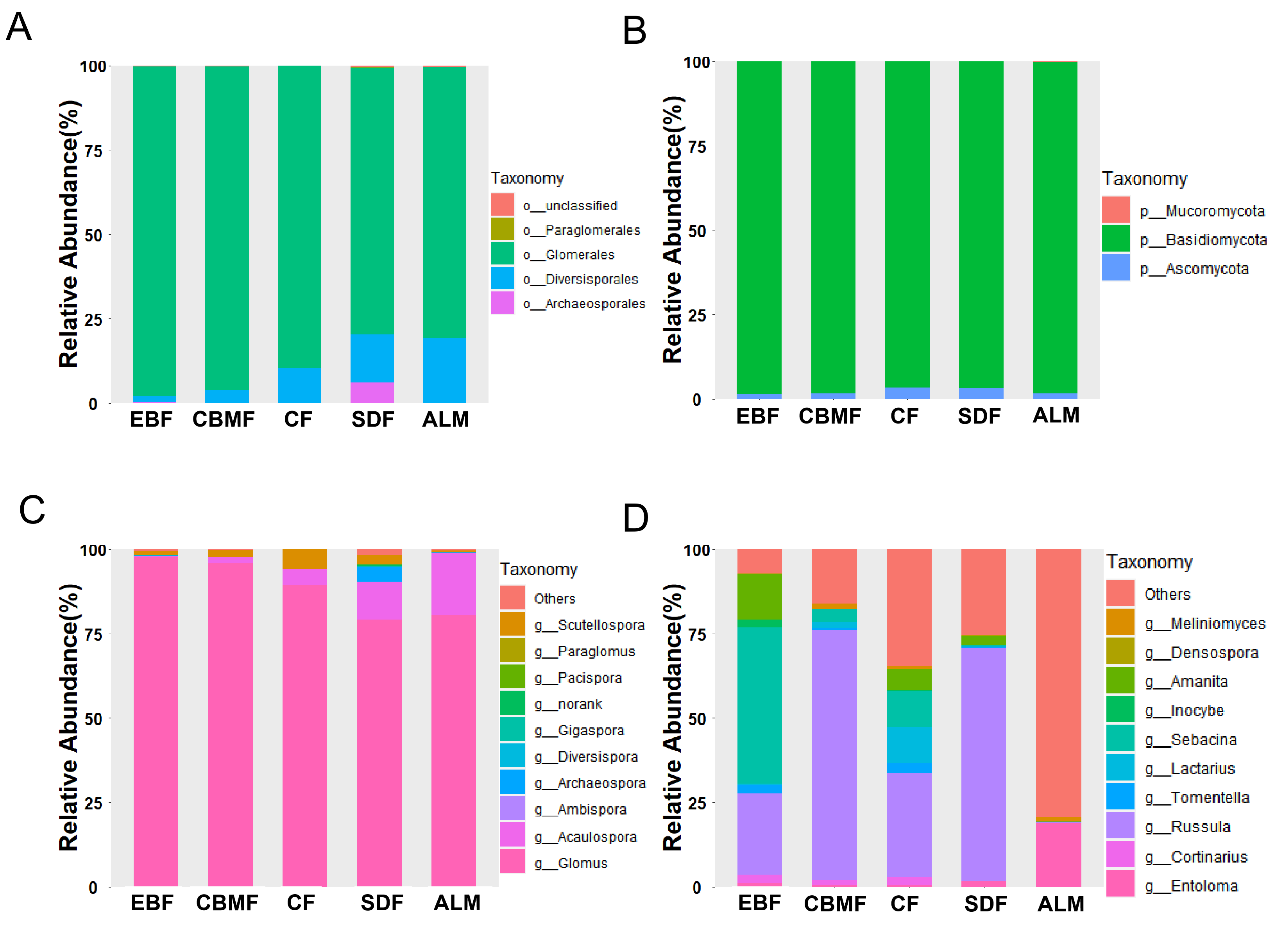


**Figure S1:** Species composition of arbuscular mycorrhizal fungi in order level (**A**) and genus level (**C**) at five elevations. Species composition of ectomycorrhizal fungi in phylum level (**B**) and genus level (**D**) at five elevations.


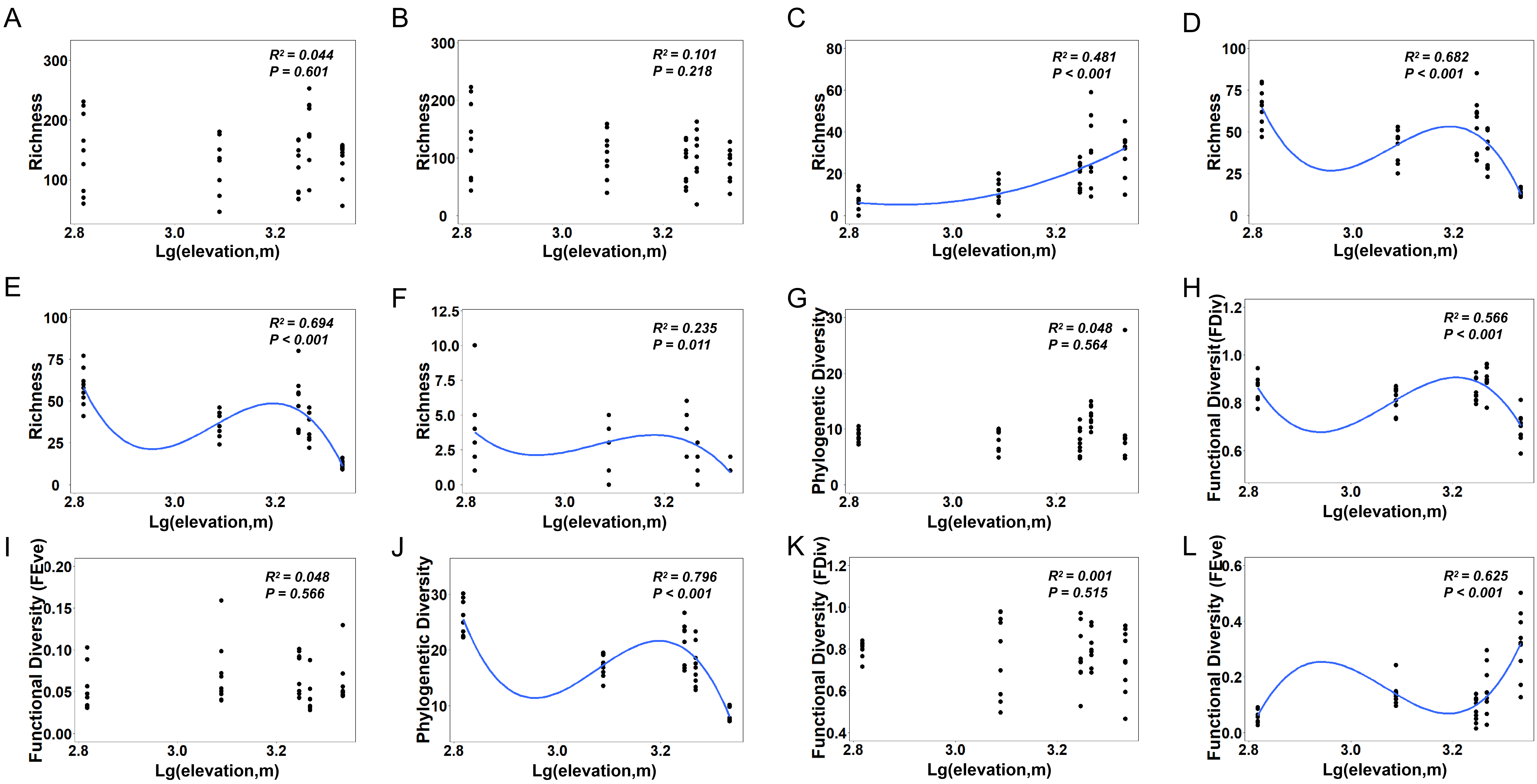


**Figure S2:** The relationship between mycorrhizal fungal diversity and elevation. Richness of arbuscular mycorrhizal fungi (**A**), Glomerales (**B**), Diversisporales (**C**), ectomycorrhizal fungi (**D**), Basidiomycota (**E**) and Ascomycota (**F**). Phylogenetic diversity of arbuscular mycorrhizal fungi (**G**) and ectomycorrhizal fungi (**J**). Functional divergence of arbuscular mycorrhizal fungi (**H**) and ectomycorrhizal fungi (**K**). Functional evenness of arbuscular mycorrhizal fungi (**I**) and ectomycorrhizal fungi (**L**).

FDiv: functional diversity; FEve: functional evenness.


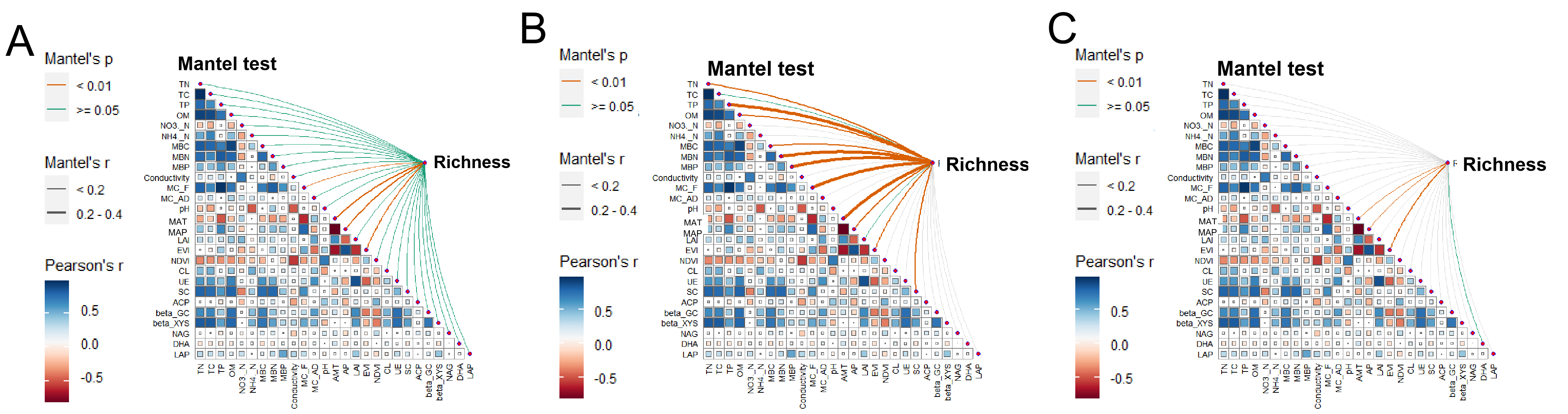


**Figure S3:** Effects of environmental factors on arbuscular mycorrhizal fungal and ectomycorrhizal fungial diversity. Richness of Diversisporales (**A**), Basidiomycota (**B**) and Ascomycota (**C**).

TC: total carbon; TN: total nitrogen; TP: total phosphorus; OM: organic matter; MBC: microbial biomass carbon; MBN: microbial biomass nitrogen; MBP: microbial biomass phosphorus; NO3._N: nitrate nitrogen; NH4._N: ammonium nitrogen; EC: electric conductivity ;MC_F: fresh soil moisture content; MC_AD: air-dried soil moisture content; MAT: mean annual temperature; MAP: mean annual precipitation; CL: cellulase; UE: urease; SC: sucrase; ACP: acid phosphatase; β-GC: β-1,4-glucosidase; DHA: dehydrogenase; NAG: β-1,4-N-acetylglucosaminidase; β-XYS: β-xylosidase; LAP: Leucine aminopeptidase.


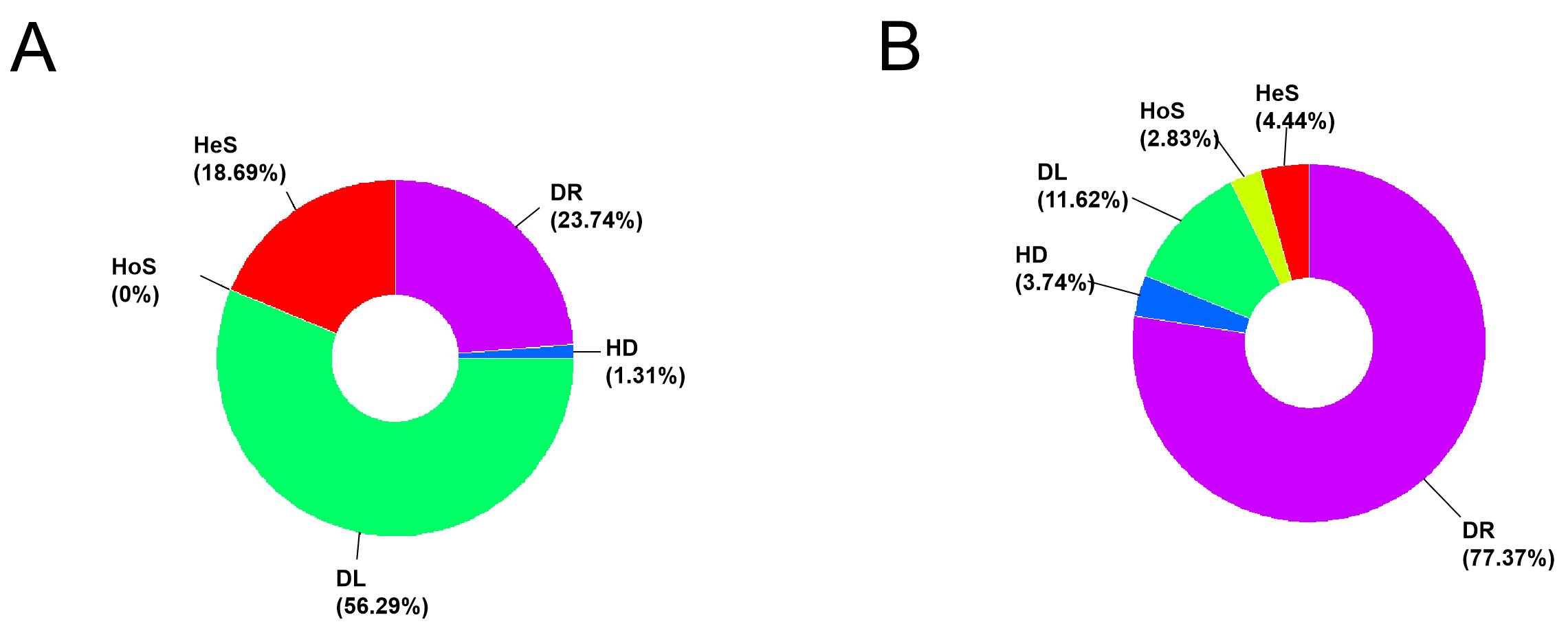


**Figure S4:** Relative importance of different ecological processes in mycorrhizal fungal community assembly. Total arbuscular mycorrhizal fungal community (**A**) and total ectomycorrhizal fungal community (**B**).

HeS: heterogeneous selection; HoS: homogeneous selection; DL: dispersal limitation; HD: homogenizing dispersal; DR: drift.


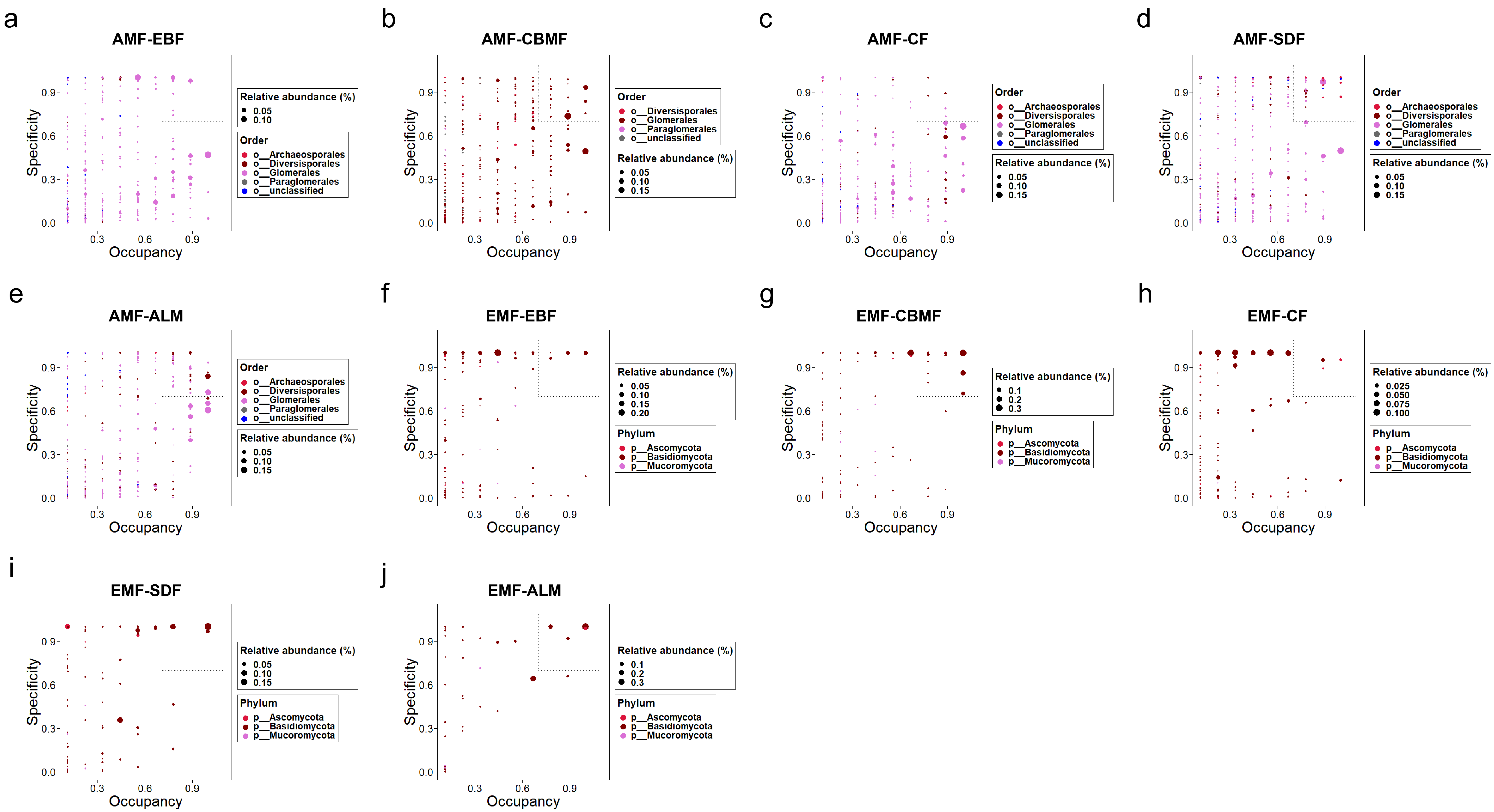


**Fig. S5: Distribution of the 500 most abundant operational taxonomic units (OTUs) in SPEC-OCCU plots.** Composition of arbuscular (a-e) and ectomycorrhizal (f-j) fungal communities across different forest ecosystems, including evergreen broad-leaved, coniferous and broad-leaved mixed, coniferous, subalpine dwarf, and alpine meadow forests. AMF: arbuscular mycorrhizal fungi; EMF: ectomycorrhizal fungi; EBF: evergreen broad-leaved forest; CBMF: coniferous and broad-leaved mixed forest; CF: coniferous forest; SDF: subalpine dwarf forest; ALM: alpine meadow.


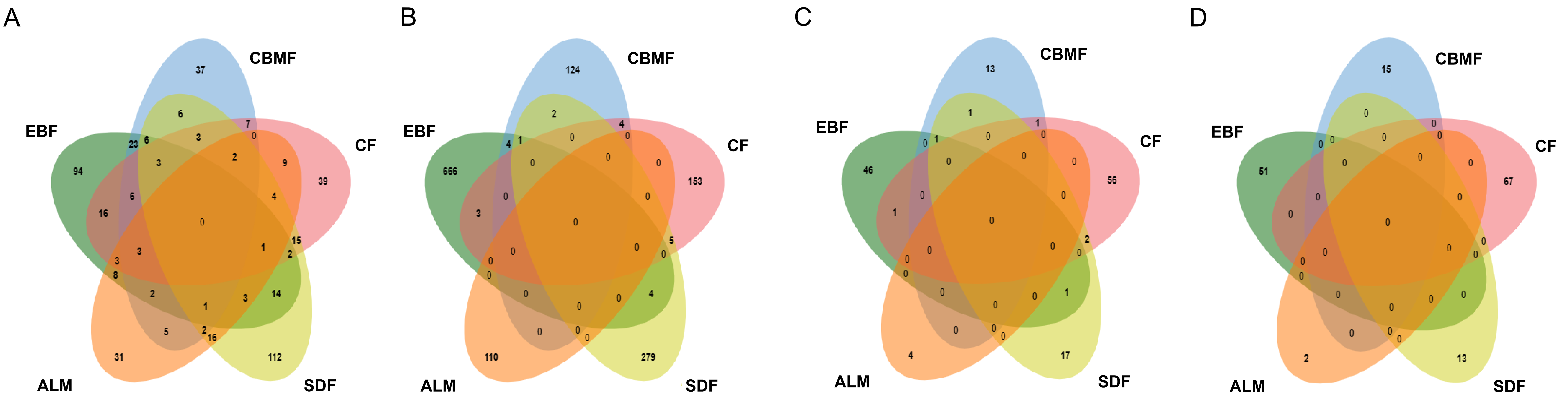


**Figure S6:** The Venn diagrams display the sharing edges and points of mycorrhizal fungi at 5 elevations. Edges (**A**) and points (**B**) of arbuscular mycorrhizal fungi, edges (**C**) and points (**D**) of ectomycorrhizal fungi.
